# Relationship between Self-Efficacy and Headache Impact, Anxiety, and Physical Activity Levels in Patients with Chronic Tension-Type Headache: An Observational Study

**DOI:** 10.1155/2022/8387249

**Published:** 2022-09-06

**Authors:** Ángel González de la Flor, Guillermo García Pérez de Sevilla, Diego Domíngez Balmaseda, Daniel Martín Vera, María Montero Martínez, Jose Ángel Del Blanco Muñiz

**Affiliations:** ^1^Faculty of Sport Sciences, Universidad Europea de Madrid, Madrid, Spain; ^2^Faculty of Biomedicine, Universidad Europea de Madrid, Madrid, Spain

## Abstract

**Background:**

Chronic tension-type headache is the primary headache with the highest prevalence. The present study is aimed at analyzing the associations between patient self-efficacy and headache impact with pain characteristics, kinesiophobia, anxiety sensitivity, and physical activity levels in subjects with chronic tension-type headache.

**Materials and Methods:**

An observational descriptive study was carried out. A total sample of 42 participants was recruited at university environment with diagnosis of tension-type headache. Headache characteristics (frequency, intensity, and duration), physical activity levels, pain related-self-efficacy, kinesiophobia, anxiety sensitivity, and headache impact were measured.

**Results:**

The HIT-6 (61.05 ± 6.38) score showed significant moderate positive correlations with the ASI-3 score (17.64 ± 16.22; *r* = 0.47) and moderate negative correlations with the self-efficacy in the domains of pain management (31.9 ± 10.28; *r* = −0.43) and coping with symptoms (53.81 ± 14.19; *r* = −0.47). ASI-3 score had a negative large correlation with self-efficacy in the domains of pain management (*r* = −0.59), physical function (53.36 ± 7.99; *r* = −0.55), and coping with symptoms (*r* = −0.68). Physical activity levels showed positive moderate correlations with the self-efficacy in the domain of physical function (*r* = 0.41). Linear regression models determined that the self-efficacy and anxiety sensitivity with showed a significant relationship with the HIT-6 score (*R*^2^ = 0.262; *p* = 0.008) and with the ASI-3 score (*R*^2^ = 0.565; *p* < 0.001). In addition, no correlations were found between pain intensity, duration or frecuency with psychosocial factors, or headache impact.

**Conclusions:**

The present study showed that patients with chronic tension-type headache had a great negative impact on daily tasks and physical activity levels, which were associated with higher anxiety levels and lower self-efficacy.

## 1. Introduction

Headache is considered the second cause of disability worldwide in people between 10 and 24 years old and ranks fifth between 25 and 54 years, according to the 2019 Global Burden of Disease Study. Besides, this report shows how depression is ranked fourth and fifth, and anxiety sixth and fourteenth for both ranges of age, respectively [1].

According to the International Headache Society (IHS) classification (third edition), Tension-Type Headache (TTH) is considered the most common primary headache [2]. TTH lifetime prevalence rate was about 26.1% to 45% [1]. TTH affects people's daily life activities in a large number of areas, which implies an increase in stress levels, impaired cognitive capacity, and a negative impact on sleep quality [3]. People with chronic daily headaches have an extremely low quality of life in all domains except for purely physical or motor functioning, which is less affected [4]. Similarly, TTH is associated with an increased number of sick leave days, as well as with lower efficiency in working tasks [5], and a lower quality of social and family relationships [6]. These consequences seem to be directly proportional to the duration, frequency, and intensity of pain [3].

Main comorbidities linked with TTH are stress, anxiety, and depression [7]. Prevalence values of anxiety and depression in subjects with TTH are 64-90% [8]. An observational study reports that headache episode frequency has been associated with anxiety, with a significant increase in anxiety when headache frequency raises [9]. Indeed, anxiety levels have been found to be significantly higher in patients with TTH than in healthy controls [10].

Some studies evaluated kinesophobia as a factor associated with TTH, although no clear data has been drawn regarding this issue [11–13]. Kinesiophobia refers to excessive, irrational, and debilitating fear a person may suffer from physical movement and/or activity due to a vulnerability perception to experience a painful injury or reinjury [14]. This fact often leads to physical inactivity as well as an increase in pain intensity in subjects with musculoskeletal pain conditions [15]. However, no significant associations between pain intensity and decreased physical activity have been reported in patients with TTH [11–13].

In regard to physical activity levels, self-efficacy seems to have a relevant role. Self-efficacy is defined as an individual's belief in his/her capacity to produce an adequate yield in daily life activities. Self-efficacy has a direct connection with headache, and it refers to the confidence level a subject has to prevent and/or control pain episodes [16]. This feature could explain why subjects with the same pain intensity levels have different levels of disability in their daily lives, since population groups with lower self-perceived ability feel they are unable to prevent or control headache attacks [17]. In fact, self-efficacy helps to improve adherence to behavioral interventions [16], and consequently, this factor should be addressed in therapeutic interventions [17].

A number of neuroimaging studies have demonstrated that morphological changes in corticolimbic structures and emotional systems are associated with persistent pain. Patients with chronic pain conditions show reductions in gray matter volume in the hippocampus and amygdala. Given the functions of these two regions, this reduction suggests that the development of chronic pain may be correlated with emotional and cognitive changes [18].

In summary, functional and structural changes in the corticolimbic system and corticolimbic interactions in patients with chronic pain can contribute to emotional and cognitive problems [19].

To date, few studies have been conducted with patients with TTH that report a broad analysis of the associated psychosocial aspects, analyzing their impact on the severity of this pathology [20–22]. In addition, TTH is common among people who spend much sitting time at work, like university employees [23, 24], and the World Health Organization global action plan on workers' health establishes that lifestyle interventions should be carried out within the workplace [25].

The present study is aimed at analyzing the associations between patient self-efficacy and headache impact with pain characteristics, kinesiophobia, anxiety, and physical activity level in subjects with TTH.

## 2. Methods

An observational study following the Strengthening the Reporting of Observational Studies in Epidemiology (STROBE) guidelines [26] was conducted in patients with chronic TTH. The study protocol adhered to the principles of the 1964 Declaration of Helsinki and its subsequent clarifications and was approved by the Research Ethics Committee of the Rey Juan Carlos University of Madrid (reference number: 1802202105721).

### 2.1. Participants

The participants were university employees recruited through the occupational health unit, when they fulfilled the following criteria: (1) adults aged 18-65 years and (2) diagnosed with chronic TTH (duration > six months) by their neurologist, following the criteria of the International Headache Society classification of headaches, in its third edition [2].

### 2.2. Variables

Anthropometric variables were age in years, height in centimeters (cm), and weight in kilograms (kg). Height was measured with a measuring rod (Ano Sayol SL, Barcelona, Spain) and weight with a mechanical scale (Asimed T2, Barcelona, Spain). Body mass index (BMI) was calculated as weight (kg)/height (m^2^) following Shephard's protocol [27].

#### 2.2.1. Physical Activity Levels

The level of physical activity was measured using the exercise habits registered with the IPAQ short-form questionnaire (International Physical Activity Questionnaire) of 7 days was used, validated, and adapted to Spanish [28, 29]. The questionnaire provides information on the estimated energy expenditure in 24 hours, by calculating the metabolic equivalents per task per minute per week (METs/minute/week), establishing a classification based on the standard values (Hagströmer et al., [30]). The questionnaire also evaluates sports experience and weekly training time, as well as physical activity in different areas of daily life such as activities at home or sedentary time [30, 31].

#### 2.2.2. Headache Characteristics

Headache duration (hours/day), intensity (from 0 (no pain at all) to 10 (the worst pain ever possible), and frequency (episodes per month) were measured following previous research [32].

#### 2.2.3. Headache Impact Test (HIT-6)

The Headache Impact Test (HIT-6) measures the impact that headaches have on daily activity tasks. Regarding the severity of the impact, ≥60 means very severe impact; 56-59, significant impact; 50-55, moderate impact; and ≤49, little impact [33].

#### 2.2.4. Tampa Scale for Kinesiophobia (TSK-11):

The Tampa Scale for Kinesiophobia (TSK) is one of the most widely used measures to assess pain-related fear in patients with pain. Factor analysis reveals a 2-factor model of 11 elements replicated in both samples, called TSK-11. The instrument shows good reliability (consistency and internal stability) and validity (convergent and predictive), with the advantage of brevity. Evidence is provided on the discriminant validity between both TSK factors (called Activity and Harm Avoidance). TSK-11 was validated by Gómez-Pérez et al. [34]. Items on the TSK-11 are scored from 1 (strongly disagree) to 4 (strongly agree). Therefore, total TSK-11 scores range from 11 to 44 points, with higher scores indicating greater fear of pain, movement, and injury. A score ≤ 28 is considered low kinesophobia; 29-35, moderate kinesophobia; and ≥36, high kinesophobia.

#### 2.2.5. Anxiety Sensitivity Index-3 (ASI-3):

The Anxiety Sensitivity Index-3 (ASI-3) measures the dispositional tendency to fear the somatic and cognitive symptoms of anxiety due to a belief that these symptoms may be dangerous or harmful. ASI-3 is a new 18-item self-report scale designed to assess the three most replicated facets of anxiety sensitivity, the physical, cognitive, and social dimensions. For ASI-3, the study by Beghi et al. [35] was followed; the responses are scored from 0 (very little) to 4 (a lot). A higher total score indicates greater anxiety [35]. A score ≤ 10 is considered low anxiety; 11-16, moderate anxiety; and ≥17, high anxiety.

#### 2.2.6. Chronic Pain Self-Efficacy Questionnaire:

The Chronic Pain Self-Efficacy Questionnaire, which assesses the patient's belief about their ability to perform certain activities, was originally developed by Anderson [36] and validated and translated into Spanish by Martín-Aragón [37]. This validation has been established as a reliable and valid instrument to assess self-efficacy expectations regarding the control of chronic benign pain, in which 19 questions were asked regarding three domains of self-efficacy: pain management, physical functioning, and coping with symptoms. Each item is answered on a scale of 0 to 10 under, where 0 means “not completely confident” and 10 “completely confident.” Higher scores indicate greater levels of confidence in dealing with pain.

### 2.3. Statistical Analysis

The Shapiro-Wilk test was employed to assess the normality [38]. A descriptive analysis was developed for all the subjects using the mean ± standard deviation (SD). In addition, to analyze the relationship between continuous variables, Spearman's correlation test and Pearson's correlation test were performed for the nonparametric and parametric variables, respectively. The magnitudes of correlation (positive and negative) between continuous variables were qualitatively interpreted using the following criteria: trivial (*r* ≤ 0.1), small (*r* = 0.1–0.3), moderate (*r* = 0.3–0.5), large (*r* = 0.5–0.7), very large (*r* = 0.7–0.9), and almost perfect (*r* ≥ 0.9) [39]. After Bonferroni's correction was applied, the statistical significance was set at an alpha level of <0.0056, as 9 comparisons were made. A multiple linear regression was performed using the force-entry method and the *R*^2^ change coefficient to state the quality adjustment. HIT-6 and ASI-3 were considered dependent variables, and self-efficacy was considered an independent variable. Graphs of standardized predicted value against standardized residuals were analyzed to assess linearity and homoscedasticity. The multicollinearity was assessed by VIF and tolerance statistics. Finally, a multiple linear regression was performed among variables that already showed significant correlations. The statistical significance was set at an alpha level of <0.05. All analyses were conducted using IBM SPSS for Windows (version 25, IBM Corporation, Armonk, New York).

## 3. Results

Adults (*N* = 42) with chronic tension-type headache were analyzed. Most of the participants were female (76%) and had a healthy body weight and low-moderate physical activity levels.

Most of the participants had long-lasting high intensity headaches.

Concerning the impact that headaches have on daily activity tasks, 83% of the participants reported a very severe or significant impact, while most of the participants reported a low kinesophobia. However, most of the participants had high (36%) or moderate (24%) anxiety levels. Regarding self-efficacy, the participants showed a high score in the physical function domain, but moderate scores in pain management and coping with symptoms.

Table 1 shows the mean scores and standard deviation of the headache's characteristics and the scores of the TSK-11, Chronic Pain Self-Efficacy Questionnaire, ASI-3, and HIT-6.

### 3.1. Correlations between the Continuous Variables

The HIT-6 score showed moderate positive correlations with the ASI-3 score (*r* = 0.47; *p* = 0.002) and moderate negative correlations with the self-efficacy in the domains of pain management (*r* = −0.43; *p* = 0.002) and coping with symptoms (*r* = −0.47; *p* = 0.005) (Table 2).

In addition, the ASI-3 score had a negative large correlation with self-efficacy in the domains of pain management (*r* = −0.59; *p* < 0.001), physical function (*r* = −0.55; *p* < 0.001), and coping with symptoms (*r* = −0.68; *p* < 0.001) (Table 2).

Physical activity levels showed positive moderate correlations with the self-efficacy in the domain of physical function (*r* = 0.41; *p* = 0.005) (Table 2). Finally, no associations were found between psychosocial factors and headache impact with pain duration, intensity, or frequency (Table 2).

### 3.2. Multivariate Predictive Analysis of Headache Impact and Anxiety

Regarding the multivariate regression analysis, the linear regression model determined significant differences (*p* < 0.05) for headache impact and anxiety sensitivity. Furthermore, self-efficacy and anxiety sensitivity (predictors) showed a significant relationship with the HIT-6 score (*R*^2^ = 0.262; *p* = 0.008) and self-efficacy with the ASI-3 score (*R*^2^ = 0.565; *p* < 0.001) (Table 3).

## 4. Discussion

The present study was aimed at examining the associations between patient self-efficacy and headache impact with pain characteristics, kinesiophobia, anxiety, and physical activity level in middle-aged individuals with TTH. Our hypothesis, based on the belief that larger anxiety or kinesiophobia levels, lower physical activity level, and lower self-efficacy, would be related to greater pain, and higher headache impact was partially supported. Lower self-efficacy and higher anxiety were associated with higher headache impact. In addition, this study identified that higher physical activity levels were related with higher self-efficacy.

Pain is complex and can rarely be explained purely by a single variable. However, the present study demonstrated the importance of considering self-efficacy when evaluating pain in patients with TTH. In fact, both lower self-efficacy and higher anxiety were related to higher headache impact in participants with TTH. Indeed, HIT-6 questionnaire score was >60, which is considered a severe impact of headaches on patients' quality of life. This finding matched with other research groups with wider study populations and similar aged-based features [40, 41].

Self-efficacy is considered a core component in self-management, yet there is a lack of knowledge about the association between self-efficacy and health-related outcomes in patients with TTH. Low self-efficacy is related to a variety of poor outcomes in both nonsurgical management and postoperative rehabilitation of musculoskeletal conditions [42–44]. Several studies have shown that evidence-based interventions can improve self-efficacy and self-management [45–47]. In this line, physical activity is a potential self-management treatment and has a positive impact on physical function and disease-related symptoms such as pain [48]. Additionally, according to Varkey et al. [49], physical inactivity is a risk factor associated with a higher prevalence of migraine. In accordance with our results, self-efficacy has been linked with physical activity [50, 51]. As noted, in previous studies, poorer pain self-efficacy predicted higher levels of anxiety [52, 53], which was associated with higher headache-related disability and frequency of episodes [9, 11, 54]. The results of the current study were consistent with these previous findings. In addition, the authors have found clinically relevant anxiety levels, according to the ASI-3 score. As previously mentioned, physical self-efficacy is associated with anxiety and it may play a role in the chronicity of headaches.

We hypothesize that pain responses such as resting and guarding the craneocervical region have been described as passive coping and viewed as reflecting patient-perceived helplessness in controlling pain or reliance on others for pain management. Resting and guarding consistently have been found to be associated with worse outcomes and thus have been considered maladaptive for chronic pain [55]. Even though findings from the present study cannot be extrapolated to treatment outcomes in patients with TTH, the characterizations of poor psychosocial health provide guidance to health professionals and should consider screening for self-efficacy and kinesiophobia in patients not responding to conservative therapeutic management.

Kinesiophobia has been studied as a clinically relevant factor linked to several extracranial pathologies such as isolated neck or shoulder pain [56]. Several studies in head-referred symptoms like migraine [57], temporomandibular disorders [58], and TTH [59] are inconsistent with our findings. Recently, one study reported that patients with chronic TTH and chronic migraine showed similar kinesiophobia scores but were significantly worse when compared to controls [13]. However, findings from the present study did not support that patients' perception and their fear of movement influence their pain experience or headache impact. Therefore, our findings disagree with the fear-avoidance model [60], which suggests that kinesiophobia is a potential psychological factor that could favor pain chronicity.

Contradicting our hypothesis, headache characteristics were not related to psychosocial factors or headache impact in people with chronic TTH. A possible explanation for this finding in that evidence regarding pain intensity, duration, or frequency in people with chronic TTH compared to asymptomatic controls is limited and conflicting [61], suggesting that resiliency factors and other potential behavioral targets such as pain acceptance can promote positive pain-related outcomes.

The present study has several limitations. Its observational nature does not allow causation, and results should be interpreted with caution. Another limitation of the study is that 76% of the sample are women and future research should determine if these results would be similar in men population. Moreover, the sample size was small, complicating output of regression models. The lack of a control group makes the results to be taken with caution. All data were self-reported and may be subject to information bias. In addition, outcomes related to depression and pain catastrophizing may be interesting, as well as other physical variables such as somatosensory, motor control, or cervical range of motion. Further studies should consider these variables to strengthen this study.

## 5. Conclusions

In summary, the present study showed that chronic TTH patients had a great negative impact on daily tasks, which was associated with higher anxiety and lower self-efficacy. In contrast, higher physical levels could enhance self-efficacy and attenuate the headache impact of this type of patients. Therefore, physical activity management and improved self-efficacy should be taken into account in chronic TTH patients.

## Figures and Tables

**Table 1:** Descriptive analysis of the variables analyzed in 42 patients with chronic tension-type headache.

Variables		Mean ± SD
Sociodemographic characteristics	Age (years)	36.69 ± 13.26
	Body mass index (kg/m^2^)	20.38 ± 3.29
Headache characteristics	Headache intensity (0 to 10)	7.14 ± 1.32
	Headaches episodes duration (hours/day)	16.18 ± 9.47
	Headaches episode frequency (times per month)	11.05 ± 9.47
QUESTIONNAIRES	TSK-11 total score ≤28 low kinesiophobia 29-35 moderate kinesiophobia ≥26 high kinesiophobia	9.00 ± 5.12 (91% low kinesophobia)
	Self-efficacy total score Range 0-190	139.07 ± 29.45
	Self-efficacy pain management Range 0-50	31.90 ± 10.28
	Self-efficacy physical functioning Range 0-60	53.36 ± 7.99
	Self-efficacy coping with symptoms Range 0-80	53.81 ± 14.19
	ASI-3 total score 0-10 low anxiety 11-16 moderate anxiety ≥17 high anxiety	17.64 ± 16.22 (36% high anxiety; 24% moderate)
	HIT-6 total score ≥60 very severe impact 56-59 significant impact 50-55 moderate impact ≤49 little impact	61.05 ± 6.38 (83% very severe or significant impact)

TSK-11: Tampa Scale for Kinesiophobia; ASI-3: Anxiety Sensitivity Index-3; HIT-6: Headache Impact Test-6.

**Table 2:** Correlations between the continuous variables.

	Headaches duration	Headache intensity	Physical activity levels	Self-efficacy coping with symptoms	Self-efficacy physical function	Self-efficacy pain management	TSK-11	ASI-3	HIT-6
Headaches frequency	-0.264	-0.234	0.277	-0.001	0.008	0.197	0.091	-0.016	0.141
Headaches duration^†^		-0.342	-0.255	0.089	-0.148	-0.027	-0.108	-0.041	-0.007
Headache intensity^†^			-0.172	0.291	-0.171	0.030	-0.278	-0.211	-0.105
Physical activity levels^†^				0.080	0.412^∗^	0.264	0.204	-0.052	0.047
Self-efficacy copying with symptoms					0.678	0.536	-0.210	-0.688^∗^	-0.471^∗^
Self-efficacy physical function^†^						0.734	-0.037	-0.554^∗^	-0.290
Self-efficacy pain management							-0.208	-0.668^∗^	-0.425^∗^
TSK-11^†^								0.095	0.048
ASI-3^†^									0.493^∗^

^∗^Significance level was set at *p* < 0.0056; ^**†**^Spearman's correlation was realized.

**Table 3:** Multivariate predictive analysis of headache impact and anxiety sensitivity.

Parameter (Dependent variables)	Model (Independent variables)	*R* ^2^ change	Beta value	*P* value
HIT-6	Self-efficacy coping with symptoms		-0.246	0.304
	Self-efficacy pain management	—	-0.065	0.780
	ASI-3	—	0.254	0.214
		0.262		0.008
ASI-3	Self-efficacy coping with symptoms		-0.352	0.049
	Self-efficacy pain management	—	-0.160	0.407
	Self-efficacy physical function	—	-0.322	0.053
		0.565		<0.001

## Data Availability

The data presented in this study are available on request from the corresponding author.

## Supplementary Materials

Supplementary Materials The questionnaires used in the present study were the TSK-11 to assess the kinesiophobia, HIT-6 for headache impact, chronic pain self-efficacy scale, ASI-3 Index to assess anxiety sensitivity, and IPAQ to evaluate the physical activity levels of the participants.
