# Supplementary material for: Relationship between Self-Efficacy and Headache Impact, Anxiety, and Physical Activity Levels in Patients with Chronic Tension-Type Headache: An Observational Study
Source: Behav Neurol. 2022 Sep 6;2022:8387249. doi: 10.1155/2022/8387249 (PMC9470367; doi:10.1155/2022/8387249)

**SUPPLEMENTARY FILES**

The questionnaires used in the present study were the TSK-11 to assess the kinesiophobia, HIT-6 for headache impact, Chronic pain self-efficacy scale, ASI-3 Index to assess anxiety sensitivity and IPAQ to evaluate the physical activity levels of the participants.

**TSK-11 (TAMPA SCALE FOR KINESIOPHOBIA SHORT-VERSION)**

**To complete, please choose one option for each item.**

| **Strongly disagree (1)** | **Disagree (2)** | **Agree (3)** | **Strongly agree (4)** |
| --- | --- | --- | --- |

1. I’m afraid that I might injure myself if I exercise
2. If I were to try to overcome it, my pain would increase
3. My body is telling me I have something dangerously wrong
4. People aren’t taking my medical condition seriously enough
5. My accident has put my body at risk for the rest of my life
6. Pain always means I have injured my body
7. Simply being careful that I do not make any unnecessary movements is the safest thing I can do to prevent my pain from worsening
8. I wouldn’t have this much pain if there weren’t something potentially dangerous going on in my body
9. Pain lets me know when to stop exercising so that I don’t injure myself
10. I can’t do all the things normal people do because it’s too easy for me to get injured
11. No one should have to exercise when he/she is in pain

TOTAL SCORE:

**HIT-6 (HEADACHE IMPACT TEST)**

HIT is a tool used to measure the impact headaches have on your ability to function on the job, at school, at home and in social situations.Your score shows you the effect that headaches have on normal daily life and your ability to function. HIT was developed by an international team of headache experts from neurology and primary care medicine in collaboration with the psychometricians who developed the SF-36® health assessment tool. This questionnaire was designed to help you describe and communicate the way you feel and what you cannot do because of headaches.

To complete, please circle one answer for each question.

1. When do you have headaches, how often is the pain severe?

| Never | Rarely | Sometimes | Very often | Always |
| --- | --- | --- | --- | --- |

1. How often do headaches limit your ability to do usual daily activities including household work, work, school, or social activities?

| Never | Rarely | Sometimes | Very often | Always |
| --- | --- | --- | --- | --- |

1. When do you have a headache, how often do your wish you could lie down?

| Never | Rarely | Sometimes | Very often | Always |
| --- | --- | --- | --- | --- |

1. In the past 4 weeks, how often have you felt too tired to do work or daily activities because of your headaches?

| Never | Rarely | Sometimes | Very often | Always |
| --- | --- | --- | --- | --- |

1. In the past 4 weeks, how often have you felt fed up or irritated because of your headaches?

| Never | Rarely | Sometimes | Very often | Always |
| --- | --- | --- | --- | --- |

1. In the past 4 weeks, how often did headaches limit your ability to concentrate on work or daily activities?

| Never | Rarely | Sometimes | Very often | Always |
| --- | --- | --- | --- | --- |

**TOTAL SCORE:**

**CHRONIC PAIN SELF-EFFICACY SCALE**

Instructions:

Please rate how confident you are that you can do the following things. 0 = very uncertain and 100 = very certain

| 0 | 10 | 20 | 30 | 40 | 50 | 60 | 70 | 80 | 90 | 100 |
| --- | --- | --- | --- | --- | --- | --- | --- | --- | --- | --- |

HOW CERTAIN ARE YOU THAT YOU CAN…

Self-efficacy for Coping with Symptoms

1. CONTROL YOUR FATIGE
2. REGULATE YOUR ACTIVITY SO AS TO BE ACTIVE WHITHOUT AGGRAVATING YOUR PHYSICAL SYMPTOMS (E.G. FATIGUE, PAIN)
3. DO SOMETHING TO HELP YOURSELF FEEL BETTER IF YOU ARE FEELING BLUE
4. AS COMPARED TO OTHER PEOPLE WITH CHRONIC MEDICAL PROBLEMS LIKE YOURS,… MANAGE YOUR PAIN DURING YOUR DAILY ACTIVITIES
5. MANAGE YOUR PHYSICAL SYMPTOMS SO THAT YOU CAN DO THE THINGS YOU ENJOY DOING
6. DEAL WITH THE FRUSTATIONS OF CHRONIC MEDICAL PROBLEMS
7. COPE WITH MILD TO MODERATE PAIN
8. COPE WITH SEVERE PAIN

Self-efficacy for Physical Function

1. WALK 1 / 2 MILE ON FLAT GROUND
2. LIFT A 10 POUND BOX
3. PERFORM A DAILY HOME EXERCISE PROGRAM
4. PERFORM YOUR HOUSEHOLD CHORES
5. SHOP FOR GROCERIES OR CLOTHES
6. ENGAGE IN SOCIAL ACTIVITIES
7. ENGAGE IN HOBBIES OR RECREATIONAL ACTIVITIES
8. ENGAGE IN FAMILY ACTIVITIES
9. PERFORM THE WORK DUTIES YOU HAD PRIOR TO THE ONSET OF CHRONIC PAIN

Self-efficacy for Pain Management

1. DECREASE YOUR PAIN QUITE A BIT
2. CONTINUE MOST OF YOUR DAILY ACTIVITIES
3. KEEP YOUR PAIN FROM INTERFERING WITH YOUR SLEEP
4. MAKE A SMALL-TO-MODERATE REDUCTION IN YOUR PAIN BY USING METHODS OTHER THAN TAKING EXTRA MEDICATION
5. MAKE A LARGE REDUCTION IN YOUR PAIN BY USING METHODS OTHER THAN TAKING EXTRA MEDICATION


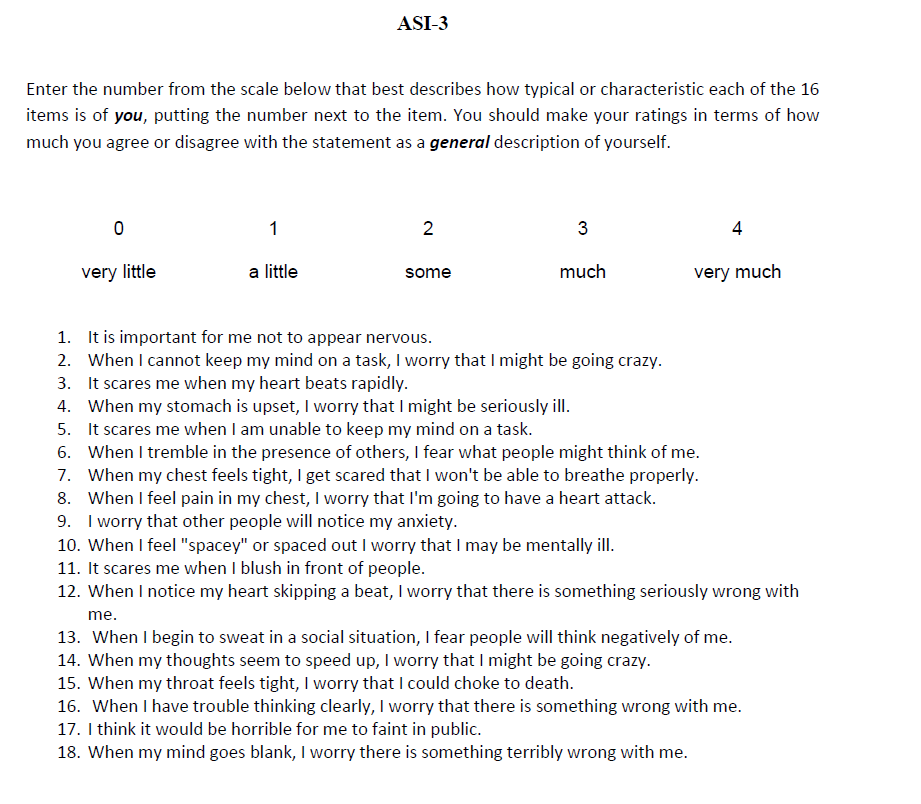


**IPAQ QUESTIONNAIRE**


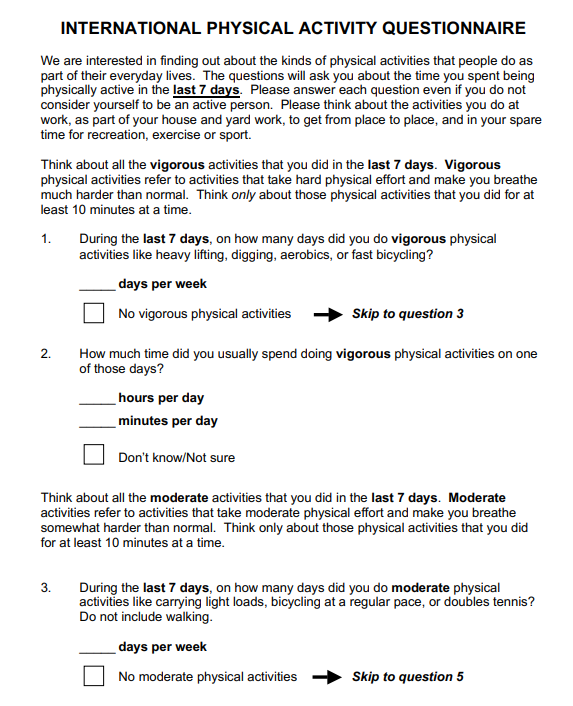


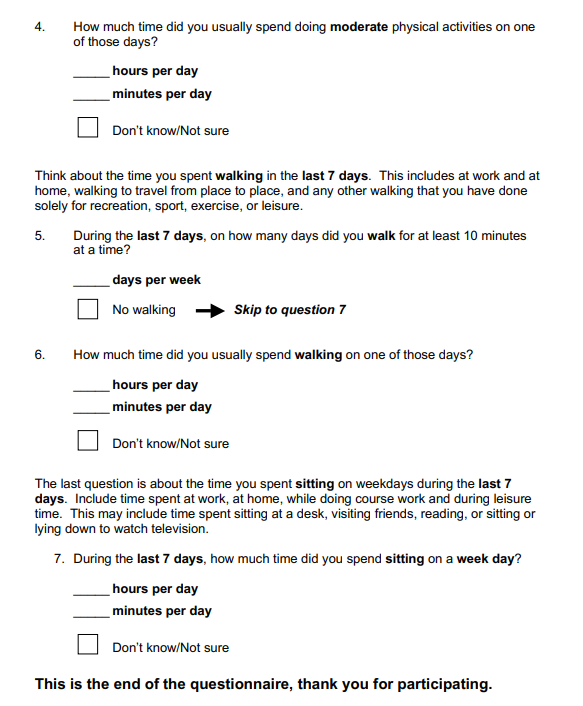

Supplement: Supplementary Materials — The questionnaires used in the present study were the TSK-11 to assess the kinesiophobia, HIT-6 for headache impact, chronic pain self-efficacy scale, ASI-3 Index to assess anxiety sensitivity, and IPAQ to evaluate the physical activity levels of the participants. [file 8387249.f1.docx]
